# Adipocyte lipolysis links obesity to breast cancer growth: adipocyte-derived fatty acids drive breast cancer cell proliferation and migration

**DOI:** 10.1186/s40170-016-0163-7

**Published:** 2017-01-13

**Authors:** Seher Balaban, Robert F. Shearer, Lisa S. Lee, Michelle van Geldermalsen, Mark Schreuder, Harrison C. Shtein, Rose Cairns, Kristen C. Thomas, Daniel J. Fazakerley, Thomas Grewal, Jeff Holst, Darren N. Saunders, Andrew J. Hoy

**Affiliations:** 1 Discipline of Physiology, School of Medical Sciences & Bosch Institute, The Hub (D17), Charles Perkins Centre, The University of Sydney, Camperdown, NSW 2006 Australia; 2 Kinghorn Cancer Centre, Garvan Institute of Medical Research, Darlinghurst, NSW 2010 Australia; 3 Centenary Institute, The University of Sydney, Camperdown, NSW 2050 Australia; 4 Sydney Medical School, The University of Sydney, Camperdown, NSW 2006 Australia; 5 Faculty of Medicine, University of Utrecht, Utrecht, The Netherlands; 6 Faculty of Pharmacy, The University of Sydney, Camperdown, NSW 2006 Australia; 7 School of Life and Environmental Sciences, Charles Perkins Centre, The University of Sydney, Camperdown, NSW 2006 Australia; 8 School of Medical Sciences, UNSW Australia, Sydney, NSW 2052 Australia

**Keywords:** Obesity, Breast cancer, Lipid metabolism, Adipocytes, Metabolic crosstalk

## Abstract

**Background:**

Obesity is associated with increased recurrence and reduced survival of breast cancer. Adipocytes constitute a significant component of breast tissue, yet their role in provisioning metabolic substrates to support breast cancer progression is poorly understood.

**Results:**

Here, we show that co-culture of breast cancer cells with adipocytes revealed cancer cell-stimulated depletion of adipocyte triacylglycerol. Adipocyte-derived free fatty acids were transferred to breast cancer cells, driving fatty acid metabolism via increased CPT1A and electron transport chain complex protein levels, resulting in increased proliferation and migration. Notably, fatty acid transfer to breast cancer cells was enhanced from “obese” adipocytes, concomitant with increased stimulation of cancer cell proliferation and migration. This adipocyte-stimulated breast cancer cell proliferation was dependent on lipolytic processes since HSL/ATGL knockdown attenuated cancer cell responses.

**Conclusions:**

These findings highlight a novel and potentially important role for adipocyte lipolysis in the provision of metabolic substrates to breast cancer cells, thereby supporting cancer progression.

**Electronic supplementary material:**

The online version of this article (doi:10.1186/s40170-016-0163-7) contains supplementary material, which is available to authorized users.

## Background

Metabolic reprogramming is considered an emerging hallmark of cancer cells and has attracted significant renewed interest both from the perspective of understanding tumorigenesis and as a potential therapeutic target [1]. An important outcome of this metabolic shift is activation of pathways that generate cellular macromolecule building blocks to support proliferation, including fatty acids and complex lipids for membrane synthesis, nucleotides for DNA/RNA synthesis, and amino acids for protein synthesis. These pathways also help cells adapt to oxidative stress and provide the energy required for biomass synthesis, migration, and invasion [2]. Much attention has centered on glucose and glutamine metabolism as substrates for these altered pathways, in particular, as precursors for de novo lipogenesis in oncogenic cell proliferation [3–5], yet the contribution of extracellular fatty acids to breast cancer metabolism is not well defined.

The nature of tumor-stroma interactions, particularly reciprocal signaling between tumor cells and fibroblasts, has been the subject of extensive study (see review [6]). However, more recently, this model has been broadened to consider the role of other stromal cell types (e.g., adipocytes) and incorporate other concepts such as reciprocal metabolic cross-talk. Martinez-Outschoorn and colleagues [7] have proposed a two-compartment energy model to describe the metabolic role of tumor stroma in cancer progression. In this model, tumors act as metabolic parasites, sequestering metabolic substrates including lactate, glutamine, and fatty acids from local/stromal sources via stimulation of catabolic pathways such as autophagy, glycolysis, and lipolysis. This is likely to be highly relevant in the breast where adipocytes, professional lipid storage cells, are the predominant cell population and are capable of secreting significant quantities of metabolic substrates such as glycerol and fatty acids. Further, there is close juxtaposition of adipocytes and breast cancer cells during early local invasion [8–10] and adipocytes are proposed to be obligate partners in cancer progression [11]. Adipocytes alter breast cancer cell growth, migration, and invasion in vitro [9, 12, 13]. However, most attention to date has focused on the production of hormones, growth factors, and cytokines by adipose tissue in tumor progression (see review [14]). Relatively, little attention has been paid to the significant potential for stromal adipocytes to provide metabolic substrates, thereby supporting breast cancer progression.

Significant epidemiological evidence suggests that obesity results in increased breast tumor size, increased rate of distant metastasis formation, and elevated mortality [15–17]. The mechanisms that underpin this relationship are yet to be defined, but in a metabolic context at least, adipocytes likely play an important role. However, the influence of obesity in modulating the effects of adipocytes on breast cancer cell behavior has received limited attention. Obesity is defined as excess accumulation of adipose tissue in an attempt to accommodate excess calories. Excess adiposity, in the form of increased triacylglycerol (TAG) levels and adipocyte dysfunction, results in increased release of fatty acids and is often associated with hyperinsulinemia, low-grade inflammation, and impaired adipokine secretion [18, 19]. Adipocytes mobilize free fatty acids from the triacylglycerol pools in a series of reactions catalyzed by adipose triglyceride lipase (ATGL), hormone sensitive lipase (HSL), and monoacylglycerol lipase (MAGL). ATGL favors TAG substrates and catalyzes the rate-limiting first step of lipolysis. In the second step, diacylglycerol (DAG) is hydrolyzed by HSL, which has broad substrate specificity and also hydrolytic activity against TAG [20]. The orchestrated activation of ATGL and HSL are required for complete lipolysis to occur in adipocytes [21].

Here, we investigated the interaction between breast cancer cells and lipid-loaded “obese” adipocytes in an in vitro model, focusing on the ability of breast cancer cells to mobilize stored energy-dense fatty acids from adipocytes and whether this energy transfer promotes breast cancer cell proliferation and migration.

## Methods

### Cell culture

MCF-7 (ERα positive, HTB-22, ATCC) and MDA-MB-231 (ERα negative, HTB-26, ATCC) human breast cancer cells were cultured in high glucose Dulbecco’s modified Eagle’s medium (DMEM) supplemented with 10% fetal calf serum (FCS; HyClone, GE Healthcare Life Sciences, USA) and 100 IU/ml penicillin and 100 IU/ml streptomycin (Life Technologies Australia Pty Ltd., Scoresby VIC, Australia). 3T3-L1 fibroblasts (CL-173, ATCC) were cultured and differentiated as described previously [22]. T47-D (HTB-113, ATCC), MDA-MB-436 (HTB-130, ATCC), MDA-MB-134 (HTB-23, ATCC), MDA-MB-175 (HTB-25, ATCC), MDA-MB-330 (HTB-127, ATCC), MDA-MB-361 (HTB-27, ATCC), MDA-MB-468 (HTB-132, ATCC), BT-483 (HTB-121, ATCC), BT-474 (HTB-20, ATCC), BT-20 (HTB-19, ATCC), and BT-549 (HTB-122, ATCC) were cultured in RPMI (1640, Gibco) with 10% (*v*/*v*) FBS, 1% (*v*/*v*) HEPES, and 0.25% (*v*/*v*) human insulin. HCC-38 (CRL-2314, ATCC), HCC-70 (CRL-2315, ATCC), HCC-1143 (CRL-2321, ATCC), HCC-1187 (CRL-2322, ATCC), HCC-1500 (CRL-2329, ATCC), and HCC1954 (CRL-2338, ATCC) were cultured in RPMI (1640, Gibco) with 10% (*v*/*v*) FBS, 1% (*v*/*v*) HEPES, and 1% (*v*/*v*) sodium pyruvate, 2%. MCF-10A (CRL-10317, ATCC) cells were cultured in HuMEC Ready medium (12752010, Invitrogen). MCF-12A (CRL-10782, ATCC) cells were cultured in DMEM/F12 (11320-033, Gibco) supplemented with 5% (*v*/*v*) horse serum, EGF, hydrocortisone, cholera toxin, and bovine insulin. All cells were grown at 37 °C in 5% CO_2_. Obese adipocytes were generated by incubating fully differentiated adipocytes in basal DMEM medium supplemented with 1 mM of a 1:2:1 palmitate (C16:0), oleate (C18:1), and linoleate (C18:2) (Sigma Aldrich, Castle Hill, NSW, Australia) for 24 h. Differentiated adipocytes were labeled as “lean.” All cell lines are validated periodically in house by Garvan Molecular Genetics using a test based on the Powerplex 18D kit (DC1808, Promega) and tested for mycoplasma every 3 months (MycoAlert™ mycoplasma detection kit, Lonza).

### Transwell co-culture experiments

Co-culture experiments used a transwell system (3.0 μm pore size, Polyester (PET) Membrane; Corning Life Sciences, Lowell, MA, USA). For experiments that assessed 3T3-L1 adipocyte biology, 5 × 10^4^ MCF-7 or MDA-MB-231 cells were seeded in the upper chamber with mature adipocytes in the bottom for the indicated times. Conversely, for experiments assessing cancer cell biology, 3T3-L1 adipocytes were grown then differentiated in the upper chamber with 5 × 10^4^ breast cancer cells in the bottom. Adipocytes or cancer cells cultured alone served as controls.

### Conditioned media generation

Conditioned media from fully differentiated 3T3-L1 adipocyte cells was generated by incubating cells for 24 h with 10% FBS in low glucose DMEM media. 10% FBS was substituted by 5% BSA when generating conditioned media from MDA-MB-231 and MCF-7 cells.

### Human primary mammary pre-adipocytes

Human breast pre-adipocytes were purchased from ZenBio Inc. (North Carolina, USA) and cultured and differentiated in proprietary media according to the manufacturer’s instructions.

### Cell proliferation and migration assays

Lentiviral particles encoding the stable GFP expression vector pLV411 [23] were packaged in HEK293T cells (CRL-3216, ATCC USA). GFP expressing MCF-7 and MDA-MB-231 cells were generated by incubation with pLV411 lentiviral supernatant using standard procedures. An appropriate viral dilution was visibly selected after serial dilution as described [24]. For proliferation assays, MCF-7^GFP^ (5 × 10^4^ cells/well) and MDA-MB-231^GFP^ (5 × 10^4^ cells/well) cells were seeded in the lower chamber and the following day, cells were co-cultured with or without either lean or obese adipocytes or incubated with or without either lean or obese adipocytes-conditioned media for 48 h. The percent cell confluence was continuously measured using IncuCyte-ZOOM according to the manufacturer’s instructions (Essen Bioscience, Millennium Science, Surrey Hills, NSW, Australia).

For cell cycle analysis, 5 × 10^5^ MCF-7 cells were cultured in either lean- or obese-conditioned media for 24 h. After incubation, cells were fixed in cold 70% ethanol at 4 °C overnight. Cells were stained with a buffer containing propidium iodide (20 μg/ml; Sigma), and cell cycle analysis was assessed as previously described [25].

MDA-MB-231 cell migration was determined in a scratch wound assay using the IncuCyte-ZOOM. MDA-MB-231 (8 × 10^4^ cells/well) cells were seeded and cultured to 100% confluence in the lower chamber in a complete medium supplemented with 10 ng/ml mitomycin-C for 2 h to inhibit cell proliferation. A uniform cell-free area was created with Essen Cell Scraper (Essen Bioscience, Millennium Science, Surrey Hills, NSW, Australia), and the relative wound density (the ratio of the occupied area to the total area of the initial scratched region) was measured using IncuCyte during co-culture with or without either lean or obese 3T3-L1 adipocytes.

### Lipid droplets visualization

Lean and obese 3T3-L1 adipocytes were seeded on glass slides, fixed with 4% PFA, and stained for Oil Red O. Lipid droplets were observed by using Leica DM4000.

### Analytical methods

Concentration of non-esterified fatty acids (NEFA-C, WAKO Diagnostics, Richmond, VA, USA) and glycerol (Free glycerol reagent, Sigma-Aldrich, Castle Hill, NSW, Australia) was determined using commercial kits. Adipocyte triacylglycerol (TAG) content was extracted using the method of Folch et al. [26] and quantified using an enzymatic colorimetric method (GPO-PAP reagent, Roche Diagnostics). Cell protein content was determined using Pierce Micro BCA protein assay (Life Technologies Australia Pty Ltd., Scoresby VIC, Australia).

### Intermediary metabolism

To assess co-culture intracellular substrate metabolism in MCF-7 and MDA-MB-231 cells, cells were incubated for 4 h with low glucose DMEM medium containing 2% BSA, 1-[^14^C]-oleate (0.5 μCi/ml, Perkin Elmer Inc., USA), 1 mM L-carnitine (Sigma), and a range of oleate (Sigma) concentrations representative of the fatty acid levels observed during co-culture (0.15 mM for isolation, 0.2 mM for lean co-culture, 0.3 mM for obese co-culture groups). Fatty acid oxidation was determined by measuring ^14^CO_2_ in the culture media by the addition of an equal volume of 1 M perchloric acid and liberated ^14^CO_2_ trapped in 1 N sodium hydroxide. Fatty acid incorporation complex lipids was assessed by a Folch extraction of cellular lipids, which were concentrated under a stream of nitrogen gas at 40 °C, resuspended in 100% ethanol, and transferred to scintillation vials to measure the ^14^C activity in the organic phase. Fatty acid uptake was calculated as the sum of ^14^CO_2_, ^14^C in the aqueous phase and ^14^C incorporation into lipid containing organic phase of cell lysates.

For the assessment of glucose and glutamine metabolism, the same media for oleate metabolism was used with the either U-[^14^C]-d-glucose or 1-[^14^C]-l-glutamine (0.5 μCi/ml, Perkin Elmer Inc., USA). Glucose and glutamine incorporated into DNA and RNA was determined by isolating DNA and RNA using QIAGEN kits according to the manufacturer’s instructions. The concentration of DNA/RNA was performed using a NanoDrop instrument. ^14^C activity in DNA and RNA was achieved by adding equal volumes of DNA/RNA to scintillation vials. The incorporation of glucose and glutamine into DNA and RNA was expressed as the ^14^C activity normalized to the DNA/RNA concentration.

### Fatty acid transfer

Fully differentiated adipocytes were incubated with 0.1 mM palmitate/oleate/linoleate (1:2:1) lean or 1 mM palmitate/oleate/linoleate (1:2:1) obese DMEM media supplemented with [9,10-^3^H(N)]-oleate (0.5 μCi/ml, Perkin Elmer Inc., USA) in 2% BSA for about 24 h. Specific activity was determined by measuring cellular TAG content (as above), and ^3^H in the TAG pool was assessed by a Folch extraction of cellular lipids followed by thin layer chromatography [27]. After incubation, adipocytes were co-cultured with either MCF-7 or MDA-MB-231 cells that were pre-seeded 1 × 10^5^ cells/ well for 24 h. MCF-7 and MDA-MB-231 cells were scraped in PBS and ^3^H activity determined by liquid scintillation counting.

### Western blot analysis

Cell lysates were prepared as previously described [28]. Cell lysates were subjected to SDS-PAGE, transferred to PVDF membranes (Merck Millipore), and then immunoblotted with antibodies for anti-ATGL (#2138), anti-HSL (#4107), and anti-GAPDH (#2118) obtained from Cell Signaling Technology (Danvers, MA), Total OxPhos Complex Kit (# 458099) from Invitrogen (Life Technologies Australia Pty Ltd), anti-14-3-3 (sc-33752) from Santa Cruz Biotech (Dallas, TX), and anti-CPT1A (#ab128568) from Abcam (Cambridge, MA).

### Gene expression survival analysis

Analysis of CPT1A gene expression, alteration frequencies, and patient outcomes (overall survival) in ER^+^ cancers (*n* = 594) from the TCGA breast cancer cohort [29] was performed using the cBioPortal for Cancer Genomics [30, 31].

### siRNA-mediated ATGL and HSL knockdown in 3T3-L1 cells

Fully differentiated 3T3-L1 adipocytes were treated with small interfering RNA (siRNA) as previously described [32]. Specifically, cells were electroporated with 200 nM scrambled (sense 5′-UUC UCC GAA CGU GUC ACG U-3′, 3′-ACG UGA CAC GUU CGG AGA A-5′) and ON-TARGETplus Non-Targeting Pool (Dharmacon) or 200 nM pooled ON-TARGETplus Mouse Lipe siRNA–SMARTpool (Dharmacon) and anti-ATGL siRNAs 1 (sense 5′-UCA GAC GGA GAG AAC GUC AUC AUA U-3′,3′-AUA UGA CGU UCU CUC CGU CUG A-5′) and 2 (5′-CCA GGC CAA UGU CUG CAG CAC AUU U-3′, 3′-AAA UGU GCU GCA GAC AUU GGC CUG G-5′) (Shanghai Genepharma). Cells were assayed 72 h following electroporation.

### Statistical analysis

Statistical analyses were performed with Graphpad Prism 7.01 (Graphpad Software, San Diego, CA). Differences among groups were assessed with appropriate statistical tests noted in the figure legends. *P* ≤ 0.05 was considered significant. Data are reported as mean ± SEM.

## Results

### Breast cancer cells stimulate lipolysis in mature 3T3-L1 adipocytes and accumulate adipocyte-derived fatty acids

**Fig. 1:**
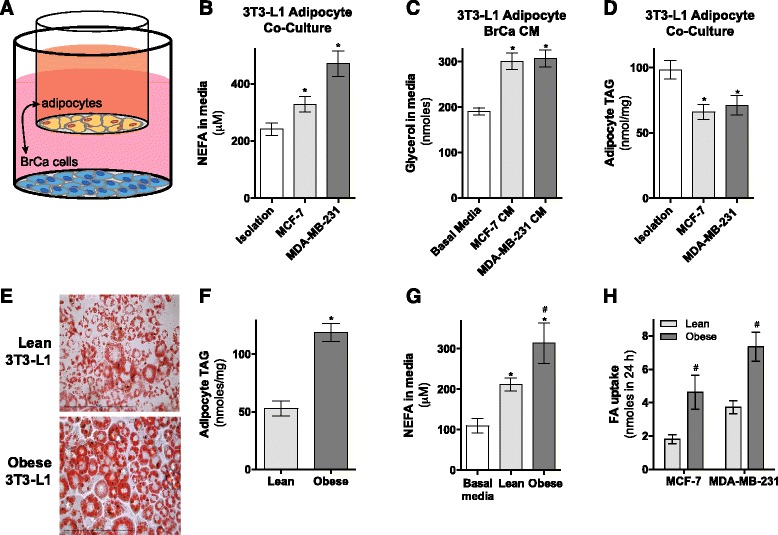
Breast cancer cells stimulate lipolysis in mature 3T3-L1 adipocytes. **a** Schematic of co-culture approach. **b** Media non-esterified fatty acids (NEFA) concentration following co-culture of 3T3-L1 adipocytes without (isolation) or with MCF-7 and MDA-MB-231 cells for 24 h (four independent experiments performed in triplicate). **c** Glycerol release from 3T3-L1 adipocytes incubated in basal media, MCF-7-conditioned media (CM), and MDA-MB-231-conditioned media at the end of 24 h incubation (seven independent experiments performed in quadruplicate). **d** 3T3-L1 adipocytes triacylglycerol (TAG) content after 48 h co-culture with or without MCF-7 or MDA-MB-231 cells (two independent experiments performed in triplicate). **e** 3T3-L1 adipocyte Oil Red O staining of lipid droplets, **f** TAG content (three independent experiments performed in triplicate), and **g** NEFA release from lean (normal media) and obese (1 mM fatty acid mixture for 24 h) adipocytes compared to basal media levels (four independent experiments performed in duplicate). **h** Transfer of adipocyte-derived ^3^H-fatty acids from lean or obese 3T3-L1 adipocytes to MCF-7 and MDA-MB-231 cells (three independent experiments performed in duplicate). Data are presented as mean ± SEM. *P ≤ 0.05 vs. controls; #*P* ≤ 0.05 vs. lean. **b**–**d** and **g** By one-way ANOVA followed by Tukey’s multiple comparisons test, **f**, **h** by Student’s *t* test

A number of studies have described reciprocal crosstalk between cancer cells and stromal adipocytes, but these have largely focused on endocrine and paracrine signaling mechanisms (see review [33]). To determine direct functional effects of breast cancer cells on adipocyte lipolysis, we used co-culture (Fig. 1a) and conditioned media approaches. Co-culture with MDA-MB-231 or MCF-7 breast cancer cells, or exposure to conditioned media (CM) from these cell lines, increased the lipolytic rate of 3T3-L1 adipocytes, as determined by non-esterified fatty acid (NEFA) and glycerol release (Fig. 1b, c, respectively). Conversely, we observed an accompanying reduction in adipocyte TAG content following co-culture with breast cancer cells (Fig. 1d). Together, these data demonstrate that breast cancer cells stimulate fatty acid mobilization from adipocyte TAG stores, consistent with previous studies [9, 34, 35].

Obesity significantly influences breast cancer behavior (see review [36]), and therefore, we extended these studies to determine whether breast cancer cell-induced fatty acid mobilization from adipocytes and transfer in vitro is enhanced in the presence of obese adipocytes. To induce obese adipocytes, we exposed 3T3-L1 adipocytes (lean) to a high-lipid environment by incubation with a physiologically relevant fatty acid mixture for 24 h [37], a similar concept to high-fat feeding rodents [38]. Adipocytes in this model displayed the cellular hallmarks of obesity, including increased lipid droplets (Fig. 1e), increased TAG content (Fig. 1f), and increased basal lipolysis rates (Fig. 1g).

To determine whether adipocyte-derived fatty acids accumulate in co-cultured breast cancer cells and assess if this is altered between cancer cells and obese adipocytes, we pulsed lean and obese adipocytes with a ^3^H-labeled fatty acid for 24 h. We then co-cultured them with breast cancer cells for a further 24 h in ^3^H-free media before measuring ^3^H-fatty acid transfer into breast cancer cells. Adipocyte-derived ^3^H-fatty acids were taken up by both MCF-7 and MDA-MB-231 cells, with MDA-MB-231 cells accumulating approximately twice the amount of fatty acids compared to MCF-7 cells (Fig. 1h). In both breast cancer cell lines, co-culture with obese adipocytes increased accumulation of adipocyte-derived ^3^H-fatty acids compared to lean adipocytes. Collectively, these data demonstrate that breast cancer cells stimulate the breakdown of adipocyte TAG stores and subsequent release of fatty acids, and these fatty acids are then transferred to adjacent breast cancer cells. Importantly, this effect is significantly enhanced in a cell culture model of obesity.

### Adipocytes alter intermediary metabolism in breast cancer cells

**Fig. 2:**
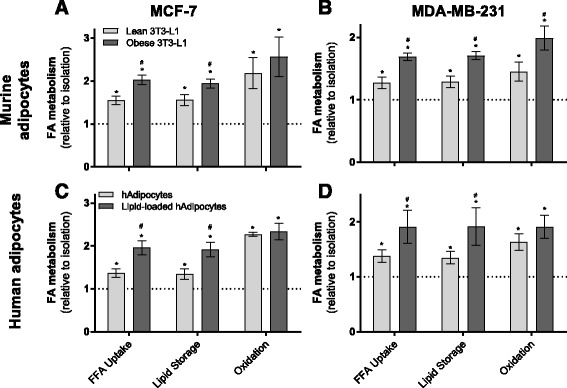
Adipocytes alter fatty acid partitioning in breast cancer cells. **a** MCF-7 cells and **b** MDA-MB-231 cells [1-^14^C]-oleate metabolism including total uptake (sum of media ^14^CO_2_, ^14^C activity in both the aqueous and organic phases of a Folch extraction), incorporation into intracellular lipids (storage), and ^14^CO_2_ generation (oxidation) after co-culture with or without 3T3-L1 adipocytes for 48 h (three independent experiments performed in triplicate). **c** MCF-7 cells and **d** MDA-MB-231 cells [1-^14^C]-oleate metabolism after co-culture with or without differentiated human primary mammary pre-adipocytes for 48 h (two independent experiments performed in duplicate). Data are presented as mean ± SEM, relative to cells in isolation (*dotted line*). *P ≤ 0.05 compared to isolation; #*P* ≤ 0.05 compared to lean by one-way ANOVA followed by Tukey’s multiple comparisons test

Next, we assessed the intracellular fate of fatty acids in breast cancer cells co-cultured with lean and obese adipocytes given the significant fatty acid transfer we observed from adipocytes to breast cancer cells (Fig. 1). Following 48-h co-culture with lean 3T3-L1 adipocytes, both MCF-7 and MDA-MB-231 cells had increased total fatty acid uptake from the media and enhanced fatty acid storage and mitochondrial oxidation (Fig. 2a, b). Co-culture with obese 3T3-L1 adipocytes had a significant additional effect on this metabolic adaptation, except for mitochondrial oxidation in MCF-7 cells (Fig. 2a, b). We observed induction of similar metabolic adaptations in breast cancer cells when co-cultured with differentiated human mammary adipocytes (Fig. 2c, d).

**Fig. 3:**
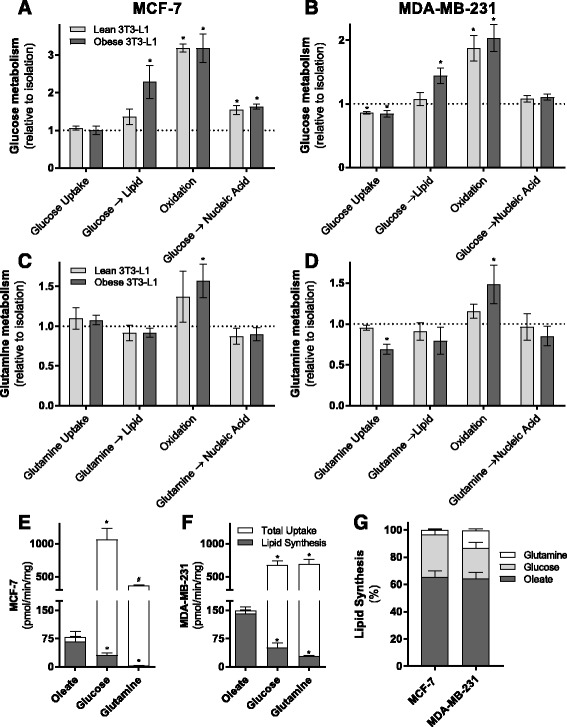
Altered glucose and glutamine metabolism in MDA-MB-231 and MCF-7 cells following co-culture with lean and obese adipocytes **a** MCF-7 cells and **b** MDA-MB-231 cells [U-^14^C]-glucose metabolism including total uptake (sum of media ^14^CO_2_, ^14^C activity in both the aqueous and organic phases of a Folch extraction), incorporation into intracellular lipids, ^14^CO_2_ generation (oxidation), and incorporation into DNA/RNA after co-cultured with or without 3T3-L1 adipocytes for 48 h (three independent experiments performed in duplicate, relative to cells in isolation (*dotted line*)). **c** MCF-7 cells and **d** MDA-MB-231 cells [1-^14^C]-l-glutamine metabolism after co-cultured with or without 3T3-L1 adipocytes for 48 h (three independent experiments performed in duplicate, relative to cells in isolation (*dotted line*)). **e**, **f** Absolute rates of ^14^C-labeled substrate incorporation into intracellular lipids (lipid synthesis) and total uptake (sum of media ^14^CO_2_, ^14^C activity in both the aqueous and organic phases of a Folsch extraction) of various substrates in MCF-7 (**e**) and MDA-MB-231 (**f**) cells and **g** percent contribution of substrates to lipid synthesis in both cell lines in the basal state (three independent experiments performed in duplicate). Data are presented as mean ± SEM. **a**–**d** *P ≤ 0.05 compared to isolation; #*P* ≤ 0.05 compared to lean by one-way ANOVA followed by Tukey’s multiple comparisons test. **e**–**f** *P ≤ 0.05 compared to oleate; #*P* ≤ 0.05 compared to glucose by one-way ANOVA followed by Tukey’s multiple comparisons test

**Fig. 4:**
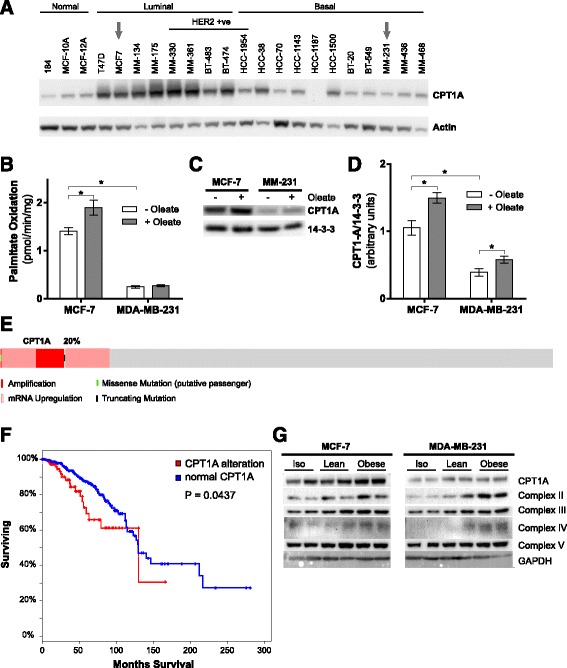
MCF-7 cells have greater CPT1 protein levels and fatty acid oxidation rates compared to MDA-MB-231 cells, and these are increased high lipid environments. **a** CPT1A expression in a range of normal, luminal, and basal breast cancer cell lines. **b** [1-^14^C]-palmitate oxidation, **c** representative immunoblots of three independent experiments, and **d** densitometric quantitation of CPT1A protein levels in MCF-7 and MDA-MB-231 cells in the basal state and after 24 h culture in 0.3 mM oleate. **e** Oncoprint output showing frequency of CPT1A alteration in a TCGA cohort of ER^+^ breast cancer (*n* = 594) (*red rectangle*, amplification; *light red rectangle*, mRNA upregulation; *black rectangle*, truncated mutation; *green rectangle*, missense mutation; *gray rectangle*, unaltered). **f** Overall survival among ER^+^ breast carcinoma TCGA cases based on CPT1A alterations. **g** Representative immunoblots of CPT1A and protein subunits in the mitochondrial complexes (complex II-30 kDa, complex III-Core protein 2, complex IV-subunit 1, and complex V-alpha subunit) in MCF-7 and MDA-MB-231 cells co-cultured with or without lean or obese 3T3-L1 adipocytes (three independent experiments performed in duplicate). Data are presented as mean ± SEM. *P ≤ 0.05 by two-way ANOVA followed by Tukey’s multiple comparisons test

Glucose and glutamine are key metabolic substrates with established roles in supporting breast cancer cell growth [39]. There is significant integration of glucose and glutamine biochemical pathways with FA metabolism, including fatty acid synthesis from these carbon sources. As such, we assessed the effects of lean or obese adipocyte co-culture on glucose and glutamine utilization by breast cancer cells. Uptake rates of glucose and glutamine were not altered in MCF-7 cells following adipocyte co-culture (Fig. 4a, c) but were reduced in MDA-MB-231 cells co-cultured with adipocytes (Fig. 4b, d). Glucose incorporation into lipid pools was enhanced in both MCF-7 and MDA-MB-231 cells co-cultured with obese 3T3-L1 adipocytes but not when co-cultured with lean adipocytes (Fig. 3a, b). No differences were observed in glutamine incorporation into lipid (Fig. 3c, d).

Co-culturing MCF-7 or MDA-MB-231 with either lean or obese 3T3-L1 adipocytes enhanced glucose oxidation in breast cancer cells by 3- and 2-fold, respectively (Fig. 3a, b). Glutamine oxidation was increased in breast cancer cells co-cultured with obese adipocytes but not in cells co-cultured with lean adipocytes (Fig. 3c, d). Incorporation of glucose carbons into cellular nucleotide pools was increased post co-culture in MCF-7 cells but not in MDA-MB-231 cells (Fig. 3a, b), with no differences observed in the incorporation of glutamine carbons into these pools (Fig. 3c, d). These data demonstrate that both lean and obese 3T3-L1 adipocytes influence multiple aspects of breast cancer cell intermediary metabolism beyond FA metabolism. These findings reinforce the importance of considering the integrated nature of metabolic biochemistry in studies of this type.

The assessment of cancer cell fatty acid metabolism is usually limited to the generation of new fatty acids from non-lipid sources such as glucose and glutamine (i.e., de novo lipogenesis). Using basal data from experiments presented in Figs. 2 and 3, we assessed the contribution of glucose, glutamine, and oleate (fatty acid) to lipid synthesis. We observed clear differences in basal substrate metabolism in the two breast cancer cell lines used in this study. MCF-7 cells take up glucose at a greater rate compared with oleate and glutamine, but oleate contributes a significantly greater amount to the cellular lipid pool compared to glucose and glutamine (Fig. 3e). Similarly, MDA-MB-231 cells take up significantly greater amounts of glucose and glutamine compared to oleate with oleate contributing a much greater amount to the lipid pool compared to glucose and glutamine (Fig. 3f). Upon further examination, oleate contributed ~65% of carbons to the total lipid pool in both cell lines with glucose providing ~30% in MCF-7 and ~20% in MDA-MB-231 cells and glutamine contributing the remainder to this pool (Fig. 3g). Furthermore, approximately 86% of oleate taken up by MCF-7 cells is stored as a lipid, compared with just 3% of glucose and 1% of glutamine with a similar pattern observed in MDA-MB-231 cells, where the vast majority of oleate (95%) is stored as lipids, compared with 8% of glucose and 4% of glutamine. Collectively, these data clearly demonstrate that lipid synthesis from glucose and glutamine carbons contribute only a small fraction (~35%) of the total lipid synthesis in the basal state. Therefore, the reported upregulation of fatty acid synthesis in breast cancer [5] is not for the sole purpose of providing the bulk mass of fatty acids.

### Adipocytes and fatty acids stimulate increased mitochondrial oxidative capacity.

Considering the consistent increase in substrate oxidation by breast cancer cells following adipocyte co-culture, we investigated the potential role of altered mitochondrial function in these cells. Carnitine palmitoyltransferase I (CPT1) is the rate-limiting enzyme in mitochondrial fatty acid oxidation, mediating fatty acid entry into the mitochondria [38]. As such, we assessed CPT1 protein expression across a panel of breast cancer cell lines representing the main molecular sub-types and observed significantly higher expression in luminal breast cancer cell lines compared to those of the basal sub-type (Fig. 4a). Consistent with this expression pattern, basal fatty acid oxidation rate is significantly higher in MCF-7 cells compared to MDA-MB-231 cells (Fig. 4b). Increasing fatty acid availability, via the addition of oleate to growth media, further enhanced fatty acid oxidation by MCF-7 cells but not MDA-MB-231 cells (Fig. 4b). This increased oxidation rate in the presence of increased oleate availability was accompanied by a marked increase in CPT1A expression in MCF-7 cells, whereas only a modest increase was observed in MDA-MB-231 cells, which have relatively lower basal CPT1A expression (Fig. 4c, d). Interestingly, analysis of 594 TCGA ER^+^ breast cancer samples showed CPT1A amplification and/or mRNA upregulation in 20% (117/594) of the ER^+^ breast cancer samples (Fig. 4e). Expression (log2) of CPT1A was significantly higher in the altered group (11.93 ± 0.97), compared to the unaltered group (10.76 ± 0.62; *P* = 1.75e^−24^). Analysis of overall survival in these 117 cases showed a significant decrease in overall survival in patients with increased CPT1A expression (*P* = 0.0439; Fig. 4f). By comparison, CPT1A expression was only upregulated in ~2% (2/107) basal-like breast cancer samples in the TCGA dataset, and showed lower overall expression in the 105 unaltered cases compared to ER^+^ samples (9.99 ± 0.80), similar to the expression levels seen in breast cancer cell lines (Fig. 4a).

Next, we extended these studies to assess the molecular changes in mitochondrial protein expression following co-culture with lean and obese adipocytes. CPT1A protein levels were increased in MCF-7 cells, but not MDA-MB-231, following co-culture with obese adipocytes compared with lean adipocytes (Fig. 4g). These data suggest that the increased fatty acid oxidation capacity in breast cancer cells stimulated by adipocyte co-culture is partly determined by CPT1A protein levels, consistent with the established role of CPT1 as the rate-limiting step in fatty acid oxidation [38]. We also observed increased expression of mitochondrial electron transport chain complex subunits in breast cancer cells following adipocyte co-culture, and this effect was further enhanced by co-culture with obese adipocytes (Fig. 4g). This observation may in part explain the observed increases in substrate oxidation in these cells following co-culture with adipocytes (Figs. 2 and 3).

### Adipocytes enhance breast cancer cells proliferation and migration

**Fig. 5:**
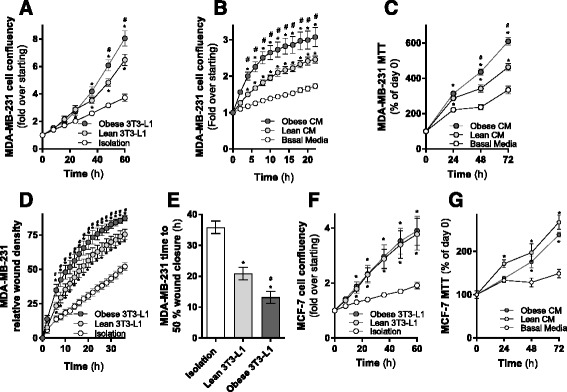
Adipocytes enhance breast cancer cells proliferation and migration rate. **a** MDA-MB-231^GFP^ cell proliferation co-cultured with or without lean or obese 3T3-L1 adipocytes (three independent experiments performed in quadruplicate). **b** IncuCyte and **c** MTT assessment of MDA-MB-231 cell proliferation cultured in conditioned media from lean or obese 3T3-L1 adipocytes (three independent experiments performed in quadruplicate). **d**, **e** Migration of MDA-MB-231 cells co-cultured with or without lean or obese 3T3-L1 adipocytes (three independent experiments performed in quadruplicate). **f** MCF-7^GFP^ cell proliferation co-cultured with or without lean or obese 3T3-L1 adipocytes (three independent experiments performed in quadruplicate). **g** MTT assessment of MCF-7 cell proliferation cultured in conditioned media from lean or obese 3T3-L1 adipocytes (three independent experiments performed in quadruplicate). Data are presented as mean ± SEM. *P ≤ 0.05 vs. controls; #*P* ≤ 0.05 vs. lean **a**–**d** and **f**, **g** by two-way ANOVA repeated measure followed by Tukey’s multiple comparisons test and **e** by one-way ANOVA followed by Tukey’s multiple comparisons test

Several studies have shown the role of endocrine and paracrine signaling mechanisms in driving adipocyte-cancer cell crosstalk [9, 12, 13]. We next sought to address the possibility that adipocyte-derived fatty acids could act as a metabolic stimulus on breast cancer cell proliferation and migration in vitro. Co-culture with lean 3T3-L1 adipocytes increased proliferation of MDA-MB-231 cells (Fig. 5a), and this was enhanced by the presence of obese adipocytes (Fig. 5a). Similar effects were observed on MDA-MB-231 cell proliferation using a complimentary conditioned media model, as reflected by increased cell confluency determined by MTT (Fig. 2b) and IncuCyte (Fig. 5c).

Increased distant metastasis is a common feature in obese women with breast cancer [40]; as such, we assessed the effect of lean and obese adipocytes on breast cancer cell migration. Co-culture with lean 3T3-L1 adipocytes increased migration of MDA-MB-231 cells, and this effect was significantly enhanced with obese adipocytes (Fig. 5d and Additional file 1: Figure S1). Time to 50% wound closure in MDA-MB-231 cells cultured in isolation was 35 h, whereas co-culture with lean adipocytes decreased this to 20 h and obese adipocytes reduced this further to 13 h (Fig. 5e). Hence, the transfer of fatty acids from adipocytes to MDA-MB-231 breast cancer cells in co-culture enhances both proliferation and migration of these cells.

Lean adipocytes increased MCF-7 proliferation during co-culture (Fig. 5f) and following exposure to adipocyte conditioned media (5G). However, we did not see any additional effects of obese adipocytes on MCF-7 cell proliferation using either co-culture or conditioned media approaches (Fig. 5f, g). Together with data presented above, these observations demonstrate clear reciprocal interactions between breast cancer cells and adipocytes, driving functional effects on both adipocyte and breast cancer cell behavior. Significantly, these effects are enhanced in the presence of obese adipocytes.

### Adipocyte ATGL and HSL are required for adipocyte-mediated effects on breast cancer cell proliferation

**Fig. 6:**
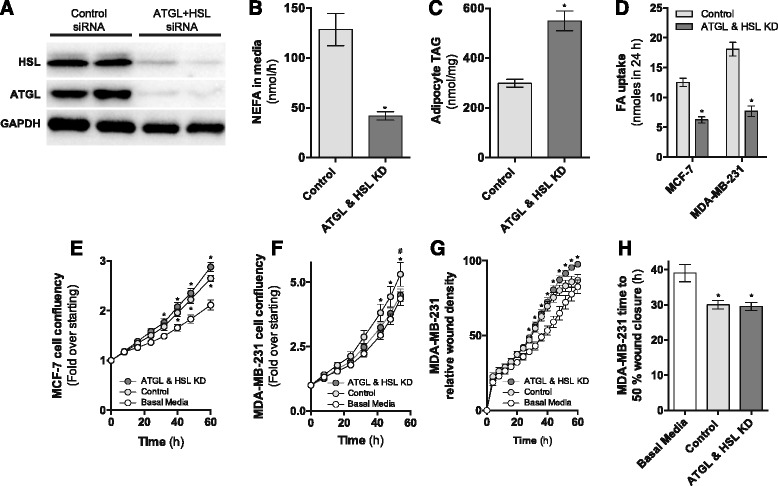
siRNA-mediated knockdown of ATGL and HSL in 3T3-L1 adipocytes. **a** Representative immunoblots of ATGL and HSL knockdown in 3T3-L1 adipocytes. Image is representative of six independent experiments. **b** NEFA secretion and **c** TAG content in 3T3-L1 adipocytes electroporated with either non-targeting (control) or LIPE (HSL) and PNPLA2 (ATGL) siRNAs (four independent experiments performed in triplicate). **d** Transfer of adipocyte-derived ^3^H-fatty acids to MCF-7 and MDA-MB-231 cells (three independent experiments performed in duplicate). **e** MDA-MB-231 and **f** MCF-7 cell proliferation co-cultured with or without ATGL/HSL knockdown 3T3-L1 adipocytes (three independent experiments performed in quadruplicate). **g**, **h** Migration of MDA-MB-231 cells co-cultured with or without ATGL/HSL knockdown 3T3-L1 adipocytes (three independent experiments performed in quadruplicate). Data are means and SEM. **b**–**d** *P < 0.05 compared to control siRNA with Student’s *t* test. **e**–**g** *P ≤ 0.05 vs. basal media, #*P* ≤ 0.05 compared to ATGL and HSL KD by two-way ANOVA repeated measures followed by Tukey’s multiple comparisons test and **h** *P ≤ 0.05 vs. basal media by one-way ANOVA followed by Tukey’s multiple comparisons test

Fatty acid release from adipocytes is dependent on sequential hydrolysis of triacylglycerol by the lipases ATGL and HSL [20]. We therefore determined the role of these lipases in mediating adipocyte-stimulated changes in breast cancer cell proliferation and migration (Fig. 5). 3T3-L1 adipocytes were transfected with siRNAs targeting ATGL and HSL, which decreased protein expression by 70 and 65%, respectively (Fig. 6a). This was accompanied by a 70% reduction in adipocyte lipolysis (Fig. 6b) and a 2-fold increase in adipocyte TAG content (Fig. 6c). Consequently, the transfer of adipocyte-derived fatty acids to MCF-7 and MDA-MB-231 cells in co-culture was attenuated by 50 and 58% respectively following siRNA-mediated knockdown of ATGL and HSL (Fig. 6d).

Proliferation of MDA-MB-231 cells grown in conditioned media from ATGL/HSL knockdown adipocytes was indistinguishable from cells grown in basal media (Fig. 6e). This indicates that the adipocyte-stimulated increase in MDA-MB-231 cell proliferation is dependent on ATGL/HSL mediated fatty acid release by adipocytes. No effect was observed on adipocyte-stimulated MCF-7 cell proliferation following ATGL/HSL knockdown (Fig. 6f). ATGL/HSL knockdown in adipocytes had a small effect on adipocyte-stimulated MDA-MB-231 cell migration at late time points, but this did not translate to differences in time to 50% wound closure (Fig. 6g, h). Collectively, these observations implicate an important role for the provisioning of adipocyte-derived fatty acids in supporting MDA-MB-231 cell proliferation.

## Discussion

Significant advances in treatment strategies have substantially improved survival rates for many subtypes of breast cancer. However, the obesity epidemic threatens to undermine these gains, with significant epidemiological evidence indicating obesity drives breast cancer progression and mortality [15–17]. The metabolic mechanisms that underpin this relationship between obesity and breast cancer are yet to be identified. In this study, we used a combination of co-culture and conditioned media approaches to demonstrate significant energy transfer from adipocytes, in the form of fatty acids, to MCF-7 and MDA-MB-231 breast cancer cells, altering intermediary metabolism, cell proliferation, and migration. These effects were enhanced by obesity in MDA-MB-231 cells and were largely dependent on ATGL/HSL-mediated fatty acid release by adipocytes.

It is well established that tumor cells can exert significant effects on adjacent stromal cells (see review [41]). More recently, the effects of cancer cells in modifying adipocyte biology have been described in ovarian cancer [42] and prostate cancer [43]. In breast cancer, several key observations have been made showing altered adipocyte function. For example, adipocytes in close proximity to tumor in primary human samples are smaller compared to more distal adipocytes [9, 35, 44] and tumor cells induce altered adipocyte gene expression and paracrine signaling factor secretion [9, 35, 44, 45]. Breast cancer cells can also significantly alter the phenotype of mature adipocytes, including reducing TAG stores [9, 34, 35]. Building on these observations, we demonstrate that this reduced TAG store is due to a breast cancer cell-stimulated increase in FA secretion (Fig. 1). The mechanisms that explain the effects of breast cancer cells on adipocytes are not well described but almost certainly involve secreted factors that alter adipocyte signaling and gene expression. For example, cancer cells stimulate expression of adipokines and adipocytokines (including IL-6, IL-1β, CCL2, CCL5, TNF-α, MCP-1, leptin), proteases, and inhibitors (e.g., MMP-11, PAI-1) [9, 35, 44, 45]. Further, pre-adipocytes in stromal vascular fraction collected from mammary fat adjacent to malignant tumors had reduced differentiation capacity compared with pre-adipocytes adjacent to benign lesions [35].

The breast cancer cell-stimulated release of significant quantities of energy dense fatty acids from adipocytes suggests they may represent a pool of metabolic substrates available to the cancer cells. This observation is consistent with breast cancer cells acting as metabolic parasites in the two-compartment energy model described above [7]. We show that adipocyte-released fatty acids are transferred to MCF-7 and MDA-MB-231 cells and that this rate of transfer is greater in the faster proliferating MDA-MB-231 cells compared with MCF-7 (Fig. 1). Further, this transfer is increased in a model of obesity (Fig. 1) and is mediated by adipocyte ATGL and HSL (Fig. 6). The mechanisms by which breast cancer cells increase fatty acid uptake in a high lipid environment are not well described. Interestingly, adipocytes also secrete insulin-like growth factor 1 (IGF-1) alongside fatty acids [46] and insulin-stimulated glucose and fatty acid uptake is phosphoinositide 3-kinase dependent in skeletal muscle and adipocytes [47, 48]. Using radiometric tracing techniques, we mapped the intracellular fate of extracellular-derived fatty acids in breast cancer cells in the presence of adipocytes. Adipocytes increased breast cancer cell fatty acid uptake, storage, and oxidation (Fig. 2). Alongside the direct utilization of fatty acids by breast cancer cells, we also observed increased conversion of glucose into lipid, and oxidation of glucose and glutamine following co-culture with adipocytes (Fig. 3). In the broader context of breast cancer cell lipid metabolism, most attention has focused on de novo lipogenesis from glucose and glutamine sources via increased expression of fatty acid synthase (FASN) and acetyl-CoA-carboxylase 1 (ACC1) [3–5]. Here, we clearly show that the predominant source for lipid synthesis by breast cancer cells is extracellular fatty acids, not glucose and glutamine (Fig. 3). This demonstrates that the widely reported increase in de novo lipogenesis by breast cancer cells does not serve as the sole source of fatty acids for membrane synthesis and other biosynthetic requirements. In fact, we show that the primary fate for extracellular fatty acids is storage as complex lipids including glycerolipids, sphingolipids, and phospholipids, consistent with a previous study showing that co-culture of MDA-MB-231 and T47D cells with primary human omental adipocytes resulted in lipid accumulation in the breast cancer cells [42]. Taken together, these observations point to an important contribution by extracellular fatty acids, including those from local adipocytes, to the intracellular lipid pools of breast cancer cells.

Breast cancer cells exposed to adipocyte conditioned media, or in co-culture with adipocytes, have previously been shown to have modified proliferation, migration, and invasion [9, 12, 13, 46, 49–52]. Similar effects have been observed in 3-D cultures [53, 54] and xenograft models [13, 55]. A range of signaling mechanisms, including IL-6, IGF-1, leptin, and adiponectin, have been proposed to explain how mature adipocytes alter breast cancer cell behavior [45, 46, 56, 57], but the role of adipocyte-derived fatty acids as direct metabolic substrates has not been investigated. Here, we show that adipocyte co-culture and conditioned media stimulate MCF-7 and MDA-MB-231 cell proliferation and migration (Fig. 5) and in MDA-MB-231 cells, this effect is dependent upon adipocyte ATGL and HSL-mediated lipolysis (Fig. 6). This was associated with increased substrate oxidation (Figs. 2 and 3), increased CPT1A expression, and increased mitochondrial electron transport chain subunit expression (Fig. 4). These observations suggest that fatty acid transfer between adipocytes and cancer cells represents a significant metabolic feature of the breast cancer microenvironment. Here, we demonstrate for the first time a direct reciprocal metabolic interaction between breast cancer cells and adipocytes. While increased CPT1A expression in ER^+^ breast cancer patients was associated with a significant decrease in overall survival, no data are available on obesity and other metabolic health parameters in this patient population. Hence, it is not possible to determine any potential contribution of obesity to increased CPT1A expression or overall survival in this cohort.

To better understand the potential metabolic role of adipocytes in mediating the effects of obesity on breast cancer cells, we generated an in vitro model of obese adipocytes and used them in our co-culture and conditioned media models. One of the major hallmarks of obesity is expanded adipose tissue and increased fatty acid availability [58, 59]. Conditioned media generated by adipocytes isolated from obese women (BMI 30–35 kg/m^2^) have been shown to increase MCF-7 proliferation compared to lean donors [46]. Further, mammary fat pad xenografts of E0771 cells had increased tumor volume in mice fed a high-fat diet to induce obesity and hyperinsulinemia compared with animals fed normal chow [51]. We show that co-culture of obese adipocytes induces a more pronounced increase in growth and migration of MDA-MB-231 cells than lean adipocytes. This was associated with increased transfer of adipocyte-derived fatty acids to MDA-MB-231 cells under obese conditions, resulting in elevated TAG synthesis and mitochondrial fatty acid oxidation. Increased transfer of adipocyte-derived fatty acids to MCF-7 cells was also observed under obese conditions, but this was not associated with a further increase in mitochondrial fatty acid oxidation, proliferation, or migration. These data highlight a potentially important role of the provision of metabolic substrates in determining the effects of adipocytes on breast cancer cell behavior in obesity.

## Conclusions

The renewed attention to understanding the unique metabolism of cancer cells has the potential to advance clinical opportunities to exploit this tumor-specific attribute beyond PET imaging and into targeted therapeutics. In this study, we have identified a significant role for fatty acids secreted from adipocytes to promote breast cancer cell growth and migration, which is exacerbated in MDA-MB-231 cells exposed to obese adipocytes. Taken together with the significant changes in adipocyte secretory profiles in obesity, the effects of obesity on breast cancer cell behavior include a direct metabolic provisioning of substrates along with the well-established paracrine and endocrine signaling effects. Hence, our data provide an additional mechanistic consideration in understanding the already well-established link between endocrine signaling and obesity, and highlight the potential for targeting lipid metabolism in breast cancer.

## Additional file

Additional file 1: Figure S1. Adipocytes enhance breast cancer cells proliferation and migration rate. Representative images of IncuCyte analysis of migration of MDA-MB-231 cells co-cultured with or without “lean” or “obese” 3T3-L1 adipocytes. (TIF 14322 kb)
