# Supplementary figures and images for: Adipocyte lipolysis links obesity to breast cancer growth: adipocyte-derived fatty acids drive breast cancer cell proliferation and migration
Source: Cancer Metab. 2017 Jan 13;5:1. doi: 10.1186/s40170-016-0163-7 (PMC5237166; doi:10.1186/s40170-016-0163-7)

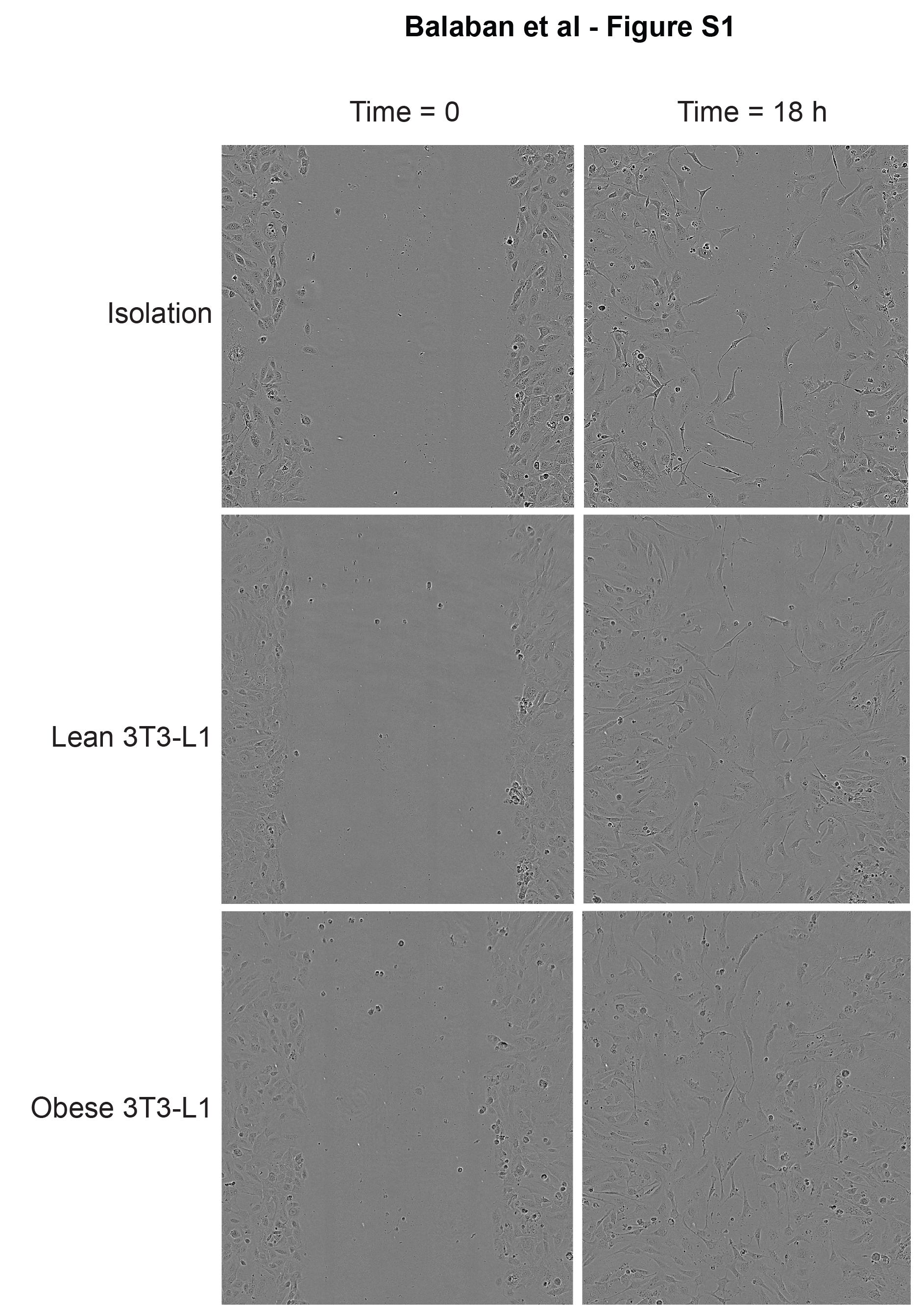

Supplement: Additional file 1: Figure S1. — Adipocytes enhance breast cancer cells proliferation and migration rate. Representative images of IncuCyte analysis of migration of MDA-MB-231 cells co-cultured with or without “lean” or “obese” 3T3-L1 adipocytes. (TIF 14322 kb) [file 40170_2016_163_MOESM1_ESM.tif]
